# Differential requirement for Dab2 in the development of embryonic and extra-embryonic tissues

**DOI:** 10.1186/1471-213X-13-39

**Published:** 2013-10-29

**Authors:** Robert Moore, Kathy Qi Cai, Wensi Tao, Elizabeth R Smith, Xiang-Xi Xu

**Affiliations:** 1 Department of Cell Biology, University of Miami Miller School of Medicine, Miami, 33136, FL, USA; 2 Graduate Program in Cell and Developmental Biology, University of Miami Miller School of Medicine, Miami, 33136, FL, USA; 3 Ovarian Cancer Program, Fox Chase Cancer Center, Philadelphia, PA19111 USA; 4 UM/Sylvester Comprehensive Cancer Center, University of Miami Miller School of Medicine, 1550 NW 10th Ave [M877], Miami, 33136, FL, USA

**Keywords:** Disabled-2 (Dab2), Primitive endoderm, Extraembryonic endoderm, Morphogenesis, Cell sorting, LDL receptor, Serum cholesterol

## Abstract

**Background:**

Disabled-2 (Dab2) is an endocytic adaptor protein involved in clathrin-mediated endocytosis and cargo trafficking. Since its expression is lost in several cancer types, Dab2 has been suggested to be a tumor suppressor. In vitro studies indicate that Dab2 establishes epithelial cell polarity and organization by directing endocytic trafficking of membrane glycoproteins. Dab2 also modulates cellular signaling pathways by mediating the endocytosis and recycling of surface receptors and associated signaling components. Previously, two independent gene knockout studies have been reported, with some discrepancies in the observed embryonic phenotypes. To further clarify the in vivo roles of Dab2 in development and physiology, we designed a new floxed allele to delete *dab2* gene.

**Results:**

The constitutive *dab2* deleted embryos showed a spectrum in the degree of endoderm disorganization in E5.5 and no mutant embryos persisted at E9.5. However, the mice were grossly normal when *dab2* deletion was restricted to the embryo proper and the gene was retained in extraembryonic tissues using Meox2-Cre and Sox2-Cre. Adult Dab2-deficient mice had a small but statistically significant increase in serum cholesterol levels.

**Conclusion:**

The study of the new *dab2* mutant allele in embryos and embryoid bodies confirms a role for Dab2 in extraembryonic endoderm development and epithelial organization. Experimental results with embryoid bodies suggest that additional endocytic adaptors such as Arh and Numb could partially compensate for Dab2 loss. Conditional deletion indicates that Dab2 is dispensable for organ development, when the vast majority of the embryonic cells are *dab2* null. However, Dab2 has a physiological role in the endocytosis of lipoproteins and cholesterol metabolism.

## Background

Mouse Disabled-2 (Dab2) was isolated as a 96 kDa phosphoprotein involved in CSF-1 signaling in macrophages, and was initially referred to as p96 [1]. A fragment of the Dab2 human cDNA was also isolated based on its frequent loss of expression in ovarian cancer, and was termed DOC-2 (Differentially expressed in ovarian carcinoma gene 2) [2]. Sequence homology suggests that the p96 protein is one of the two mammalian orthologs of the *Drosophila* Disabled gene [3]; hence, that was the origin of the naming for the neuronal expressed mammalian Dab1 [4] and the more ubiquitously expressed Dab2 [1,5].

The loss of expression of Dab2 in ovarian cancer and growth regulatory properties in cell culture studies led to the suggestion that Dab2 is a tumor suppressor in ovarian cancer [6,7]. Subsequently, loss or reduction of Dab2 expression was found in other cancer types including rat mammary tumors [8], breast cancer [9,10], colon cancer [11], esophageal cancer [12], urothelial carcinomas [13], prostate cancer [14], head and neck cancer [15], and nasopharyngeal carcinomas [16]. Mechanisms were also suggested for Dab2 in epithelial organization [10,17,18], and in the regulation of Ras/MAPK [19-22], TGF beta [15,23,24], and Wnt [25-28] signaling pathways.

Cell biology studies revealed that Dab2 is an endocytic adaptor protein [29]. Dab2 contains an N-terminal PTB domain that binds cell surface proteins with an NPXY motif in their cytoplasmic tails [30]; several motifs that bind clathrin and adaptin proteins [31]; and a C-terminal region that binds myosin VI, a directional motor protein [32,33]. Thus, Dab2 mediates the simultaneous attachment of clathrin-coated cargos containing transmembrane proteins with one or more NPXY motifs, such as the low density lipoprotein (LDL) receptor, megalin, and integrins, to the myosin motor, enabling endocytosis and directional trafficking. A role of Dab2 in endocytosis and trafficking of integrins and thus cell mobility has also been suggested [34,35]. Modulation of LDL receptor endocytosis by Dab2 has also been studied in cultured cells [36], though a role in vivo has not yet been established. The polarized trafficking of cell adhesion molecules such as integrins and E-cadherin may explain the role of Dab2 in epithelial polarity and organization [18] and trafficking of signaling surface receptors may account for its activity in modulating multiple signaling pathways [5,28,37].

To determine if any of these cellular mechanisms may be biologically relevant and significant, gene deletions in mice have been performed [17,38]. A gene replacement of *dab2* allele by betaGal-Neo led to the finding of early embryonic lethality in the knockout mice [17]. In the mutants, extraembryonic endoderm cells intermixed with ectodermal cells in the E5.5 embryos, and a visceral endoderm layer failed to develop [17,18]. The aged heterozygous mice were found to develop ovarian cysts and preneoplastic lesions in both ovaries and uteri [39]. A flox *dab2* mutation was made and the homozygous deleted mutant mice were also embryonic lethal, but the mutant embryos were found to persist to a later stage [38]. Additionally, a mosaic *dab2* deletion using a Meox2-Cre line was found to produce remarkably normal mice with minor defects in kidney function [38].

One possibility to explain the discrepancy between these two *dab2* mutant lines is that the expression of betaGal-Neo in the *dab2* mutant embryos [17] might lead a more severe phenotype. Another idea is that the *dab2* flox mutant allele [38] may produce truncated proteins from an alternative translation start site. Hence, the later knockouts may not be complete nulls and the mutant embryos may be able to persist longer.

To investigate the differences in the two knockouts and to study further the relevant biological functions and possible tumor suppressor role of Dab2, we have designed another flox *dab2* mutant allele with a more extensive exon deletion, produced the constitutive and conditional mutant mice, and examined the phenotypes. Here, we report a detailed characterization of the embryonic phenotypes, the conditional knockout mice, the mutant embryonic stem cells, and the embryoid bodies.

## Results

### Construction of floxed dab2 mutant allele and mice

**Figure 1:**
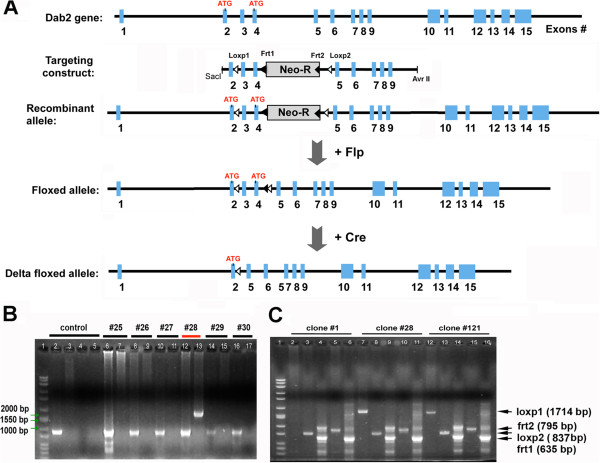
**Conditional targeting strategy and screening for homologous recombination of *dab2 *gene. (A)** Schematic illustration of *dab2* gene targeting strategy and gene deletion in mutant mice is shown. The targeting construct was made by inserting a neomycin resistance gene (Neo-R) flanked by Frt sites (closed triangles) between exon 4 and 5. LoxP sites (open triangles) were placed flanking exon 3 and 4. Correct homologous recombination of the targeting construct did not alter *dab2* gene but allowed expression of Neo-R for selection of mutant ES clones. Following selection and verification, chimeric and then germline mutant mice were made from the mutant ES cells. The Neo-R cassette was excised by crossing with Flp expression mice to generate the floxed allele. The deletion of exon 4 removed a potential alternative translation start site, indicated as “ATG”. **(B)** Examples of PCR screening assay of neomycin resistant clones (#25-30) following transfection of linearized targeting construct and drug selection. Each clone was amplified separately for wildtype (886 bp) and recombinant allele (1714 bp, as predicted). Here, clone #28 was identified as positive. In the agarose gel: lane 1: Mw markers; lane 2: control mouse DNA; lane 3: KO construct; lane 4: control WT mix, no DNA; 5: control mix for mutant, no DNA; lanes 6 to 17, amplification using DNA template from clone #25 to 30. **(C)** Examples of targeted allele in selected clones (#1, #28, and #121). Here, clone #1 was found to lack the loxP1 site, whereas clones #28 and #121 contained all components: LoxP1, 1714 bp; LoxP2, 795 bp; Frt1, 635 bp; Frt2, 837 bp; and Neo, 617 bp.

To investigate further the biological roles of Dab2 in vivo and resolve the discrepancy in the embryonic phenotypes, we designed a new *dab2* mutant using a Cre/loxP strategy. The previously reported Dab2 conditional knockout [38] was designed to delete only exon 3, resulting in a frame-shift mutant protein. However, exon 4 contains an in-frame methionine codon that may serve as an alternative translational start site and produce a truncated Dab2 mutant protein. A homologous targeting construct was prepared with loxP sites flanking both exons 3 and 4 to avoid the generation of alternative translational products (Figure 1A).

Upon transfection of ES cells with the linearized targeting construct and G418 selection, 380 independent drug-resistant clones were picked and screened for homologous recombination by PCR (Figure 1B). A total of 6 clones were identified as potentially homologous targeted lines, and 2 of the clones were characterized by PCR as containing the complete targeting construct and correct homologous replacement at both 5’ and 3’ ends (Figure 1C). These two lines of ES cells were used for blastocyst injections, chimeric mouse production, germ-line transmission, and establishment of the mutant colonies. The Neo locus was then removed by crossing with FLPeR mice [40,41] (Figure 1A). The two lines of the conditional mutant mice were bred to homozygous *dab2* flox, and the transmission of *dab2* (+/+), (+/fl), and (fl/fl) followed a Mendelian ratio (Additional file 1: Table S1 and Table S2). Homozygous flox mice exhibited wildtype characteristics with normal Dab2 expression, reproductive capability, and lifespan. We conclude that the flox alleles do not influence *dab2* gene activity and the flox mice are essentially wildtype, and the two *dab2* mutant lines are identical.

### Characterization of Dab2 constitutive null mutant embryos

The *dab2* flox mice were crossed with Meox2-Cre transgenics [42,43] to delete the *dab2* gene to generate delta flox (df) (Figure 1A). Here, we refer to the *dab2*-deleted allele in mosaic mice as “df” and constitutive null in germ-line deletion as “-”. Next, we selected both male and female progenies with the *dab2* (+/-) genotype for further matings to investigate the embryonic phenotype of *dab2* constitutive knockouts, referred to as *dab2* (-/-).

**Figure 2:**
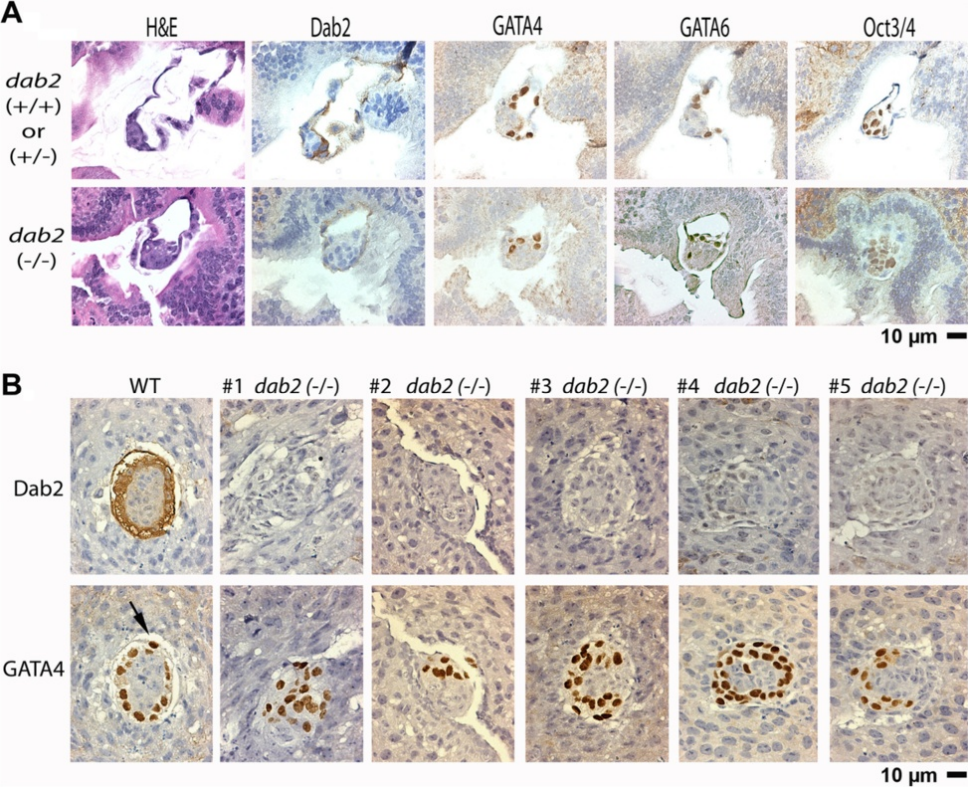
**Histological phenotypes of constitutively Dab2 null embryos at E4.5 and E5.5. (A)** Fixed and paraffin embedded E4.5 embryos *in utero* from timed matings of *dab2* (+/df) mice were sectioned and stained with H&E to identify implanted embryos. Once found, the serial sections were used for staining with Dab2 to identify *dab2* deletion mutants, adjacent sections were then stained with GATA4 and GATA6 as markers for primitive endoderm; and with Oct3/4 to identify cells of the inner cell mass. Consecutive sections of a representative Dab2-positive and a Dab2-negative (*dab2* (-/-)) E4.5 embryo are shown. **(B)** Examples of one wildtype (WT) and five Dab2-negative (*dab2* (-/-)) E5.5 embryos are shown. Dab2 staining was used to identify mutant embryos. An adjacent section was stained for GATA4 to identify the endoderm cells. The images shown were sections at or near midline (widest area) of the embryos. A spectrum of severity in disorganization of endoderm cells was seen in the *dab2* null embryos. The arrow indicates a parietal endoderm cell in the wildtype.

We initially examined embryos at E4.5 when Dab2 is expressed in the primitive endoderm [17]. At this implantation stage, *dab2* (-/-) embryos were identified by immunostaining for the absence of Dab2 protein (Figure 2). In wildtype or heterozygous blastocysts, the Gata4-, Gata6-, and Dab2-positive primitive endoderm cells exclusively located on the surface layer covering the Oct3/4-positive inner cell mass. Among 26 E4.5 embryos found following sectioning of 6 uteri, 4 implanted blastocysts were confirmed as Dab2-null based on immunostaining. In the Dab2 knockout embryos, although Gata4- and Gata6-positive primitive endoderm cells were present, the cells were not organized into a monolayer epithelium and some located deep inside the inner cell mass (Figure 2A). Thus the embryonic defect is initiated at E4.5, post differentiation but prior to morphogenesis of the primitive endoderm, although the phenotype is subtle at this stage.

By E5.5, the disorganization of the extraembryonic tissues derived from the primitive endoderm was apparent (Figure 2B). For comparison, all the Dab2-positive E5.5 embryos, of either *dab2* (+/+) or (+/-), were well structured with a Gata4- and Dab2-positive ring of visceral endoderm surrounding the epiblast (Figure 2B, WT). A thin layer of parietal endoderm was present at the outer layer (Figure 2B, WT, only one Gata4-positive nucleus is visible in this section, indicated by an arrow). Of the 65 E5.5 embryos sectioned and analyzed, 55 were Dab2-positive (either wildtype or heterozygous), and 10 were confirmed as null. In the Dab2-negative E5.5 embryos, Gata4-positive endoderm cells were misplaced and intermixed within the egg cylinder core in all mutant embryos analyzed (Figure 2B), as shown in the 5 representative examples of sections through the center of the embryos. Unlike wildtype embryos that had uniformly well patterned endoderm epithelium, all mutant embryos showed a spectrum in the degree of endoderm disorganization ranging from mild to severe (Figure 2B, mutant embryos #1-5).

Since the previously reported Dab2 mutant embryos persisted and defective embryos were observed at E9.5 [38], we set up timed matings to genotype embryos specifically at E9.5 for both *dab2* mutant lines. No dissected embryos were determined as *dab2* (-/-) in 12 timed matings (Table 1). However, a significant number of reabsorbed embryos, which are likely homozygous mutants, were observed. Thus, we conclude that the current *dab2* mutant embryos exhibit embryonic lethality at around E5.5 and no embryos can survive to E9.5, consistent with the embryonic phenotype reported for the betaGal-Neo mutant [17,18]. For E6.5 to E8.5, we observed putative mutant embryos in a various states of degeneration and re-absorption, which we found to be uninformative and have not systematically characterized them.

**Table 1:** **Genotyping of *dab2 *** **constitutive mutant embryos recovered at E9.5**

**Genotypes**				
**Lines**	**+/+**	**+/-**	**-/-**	**Reabsorbed**
**#270**	**17**	**21**	**0**	**15**
**#239**	**15**	**18**	**0**	**16**

**Note:** E9.5 embryos from matings between two lines (#270 and #239) of Dab2 mutant mice of *dab2* (+/df) parents were genotyped by PCR. In total, embryos harvested from12 pregnant females were analyzed. The number of reabsorbed embryos was noted by visual examination of the uteri.

### Characterization of dab2 conditional mutant mice

**Figure 3:**
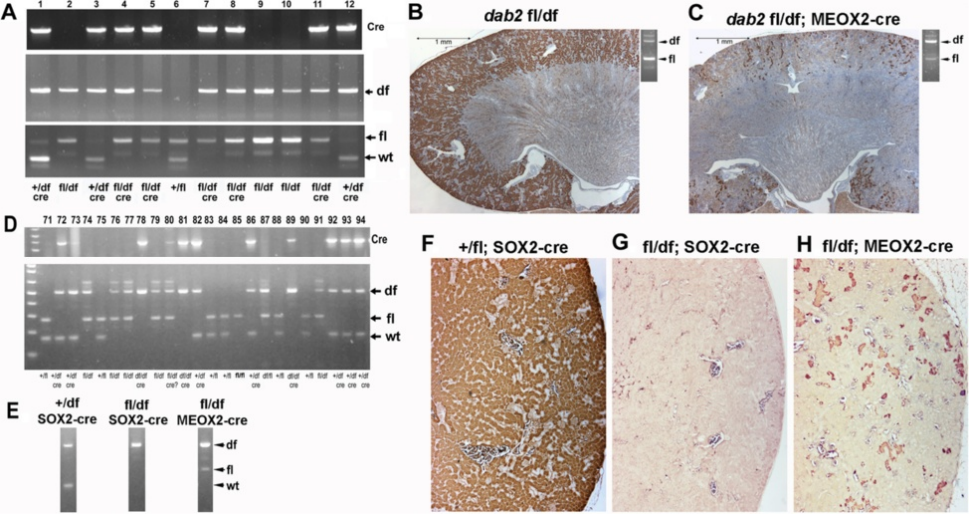
**Conditional *dab2 *deletion by Meox2-Cre and Sox2-Cre transgene.** Female *dab2* (fl/fl) mice were crossed with male *dab2* (df/+);Meox2-Cre or *dab2* (df/+);Sox2-Cre mice to produce mosaic mutant mice without deletion in the extraembryonic endoderm. Mice of around the age of 3 months were used for these analyses. **(A)** An example of PCR genotyping assay using tail DNA of the progenies (#1 to 12) generated from the cross between *dab2* (fl/fl) and *dab2* (df/+);Meox2-Cre. Specific PCR amplifications to identify the locus for cre, fl, df, and wt were performed either separately or in a combined reaction. Examples of Dab2 immunostainings in kidney of a *dab2* (df/fl) **(B)** and a *dab2* (df/fl);Meox2-Cre **(C)** mouse are shown, with corresponding PCR genotyping results shown at the upper right corner. **(D)** An example of PCR genotyping assay using tail DNA of the progenies (#71 to 94) generated from crosses between *dab2* (fl/fl) and *dab2* (df/+);Sox2-Cre. **(E)** DNA gel resolving the genotyping PCR products shows a comparison of the deletion efficiency generated by Sox2-Cre or Meox2-Cre. Corresponding Dab2 stainings show the extent of Dab2 mosaicism in the kidney sections from *dab2* (+/df) **(F)**, *dab2* (fl/df);Sox2-Cre **(G)**, and *dab2* (fl/df);Meox2-Cre **(H)** mice.

We further examined the Dab2 floxed mice using Meox2-Cre to bypass the essential function of Dab2 in extraembryonic endoderm. Cre expression initiates in the epiblast but is absent in extraembryonic tissues [44]. We generated the conditional mutants by crossing female *dab2* (fl/fl) with male *dab2* (df/+);Meox2-Cre mice. Thus, the resulting *dab2* (df/fl);Meox2-Cre mice were expected to be mosaic for deletion of *dab2* in the embryo proper. Mice genotyped as *dab2* (df/fl);Meox2-Cre were produced and exhibited no detectable developmental defects. PCR genotyping of the tail tissues indicated that 90% of the floxed alleles were deleted, detected as “df”, and only 10% of the *dab2* alleles remained intact as “fl” at around 3 weeks of age (Figure 3A). Dab2 is highly expressed in kidney [6], and immunostaining indicated kidney proximal tubular epithelial cells were evenly positive (Figure 3B). However, in the adult conditional knockout mice (around 3 months), Dab2 protein was lost in 90% or more of the cells (Figure 3C). Thus, when Meox2-Cre is used for gene deletion, substantial numbers of non-recombined cells nevertheless persist in adult mutant mice.

Subsequently, we used another Cre line, the Sox2-Cre transgenic [42], in an attempt to delete *dab2* to a greater extent. Like Meox2, Sox2 is expressed in the epiblast but not in extraembryonic tissues, and Sox2-Cre line can efficiently delete the floxed gene in the embryo proper [42,43]. The conditional mutants were generated by crossing female *dab2* (fl/fl) with male *dab2* (df/+);Sox2-Cre mice. Here, male rather than female carriers of Sox2-Cre were used to avoid a maternal effect, which, due to Cre expression in oocytes, causes non-Mendelian transmission [42,43]. We found that the *dab2* “fl” allele was consistently undetectable by PCR in tails of conditional knockouts at 3 weeks of age (Figure 3D). Comparisons of representative PCR amplifications from heterozygous, conditional knockouts using Sox2-Cre or Meox2-Cre, respectively, are shown (Figure E). The kidney from adult Sox2-Cre conditional knockouts lacked any Dab2-positive cells (Figure 3F-H). Nevertheless, *dab2* (df/fl);Sox2-Cre mice were produced at a Mendelian ratio and were devoid of any obvious developmental defects. Both male and female mutant mice were fertile and had normal breeding ability when paired with a wildtype partner (Additional file 1: Table S1 and Table S2).

**Figure 4:**
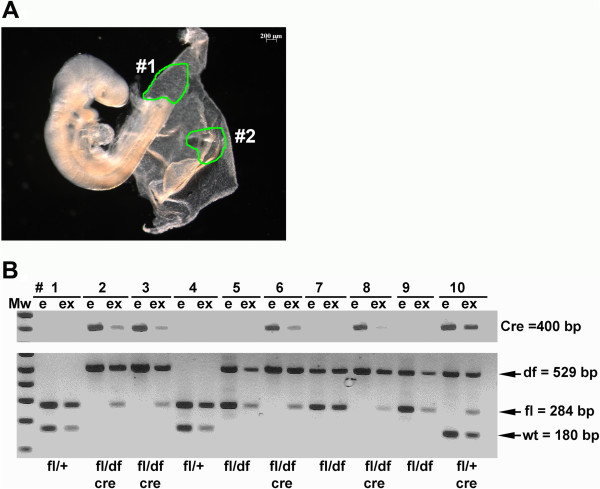
**Efficiency of *dab2 *deletion in embryonic and extraembryonic tissues by Sox2-Cre in E9.5 embryos.** E9.5 embryos from a timed mating between female *dab2* (fl/fl) and male *dab2* (df/+);Sox2-Cre were dissected and harvested for analysis. **(A)** The example shows the region dissected as embryo (#1) and extraembryonic tissue (#2). **(B)** The embryonic (e) and extraembryonic (ex) tissues were separated and used for PCR genotyping of *dab2* gene to determine the presence of Cre (400 bp), df (529 bp), fl (284 bp), and wildtype (wt, 180 bp) allele, to estimate the efficiency of *dab2* gene deletion.

To address the possibility that a minor fraction of residual Dab2-positive cells might be involved and critical for embryonic development and organogenesis, we assessed the potential presence of Dab2-positive cells in early embryos of Sox2-Cre-mediated conditional *dab2* knockout. Female *dab2* (fl/fl) were crossed with male *dab2* (df/+);Sox2-Cre mice to obtain E9.5 embryos in which the embryonic (e) and extraembryonic (ex) tissues were dissected, discretely separated, and used for PCR genotyping (Figure 4). In each embryo inheriting Sox2-cre (embryos # 2, 3, 6, 8, 10 in Figure 4), significant levels of the “fl” allele were present in the extraembryonic compartments but the allele was undetectable in the embryo proper (Figure 4). An identical conclusion was reached from analysis of the Sox2-Cre-mediated embryonic and extraembryonic *dab2* gene deletion at E10.5 (not shown). Thus, the efficiency of *dab2* gene deletion by Sox2-Cre was high, and essentially 100% in embryonic cells by E9.5. Therefore, Dab2 is dispensable for tissue formation and organogenesis within the embryo proper.

Up to now, we have characterized more than 200 progenies with a *dab2*-deleted genotype and have observed no obvious developmental phenotype. However, the aged mice exhibited an increased incidence of preneoplastic lesions, mainly in the uterus, ovary, mammary gland, and colon, similar to the previous report for the *dab2* heterozygous mice [39]. The studies of the tumor phenotypes of aged Dab2 knockout mice and in combination with p53 mutation will be reported elsewhere.

### Role of Dab2 in cholesterol metabolism in vivo

We made additional attempts to determine possible physiological phenotypes of the Dab2-deficient mice based on known cellular functions of the Dab2 protein. Dab2 is an endocytic adaptor protein for several NPXY motif-containing cell surface receptors, including lipoprotein (LDL) receptor, megalin, and integrins. Endocytosis of megalin mediated by Dab2 is thought to play a role in protein re-uptake in the proximal tubule cells of kidney, and previously it was found that excessive proteins were present in the urine from Dab2-deficient mice [38]. A mild proteinuria phenotype was also observed in the Dab2-deficient (*dab2* (df/fl);Sox2-Cre) mice in our current study.

**Figure 5:**
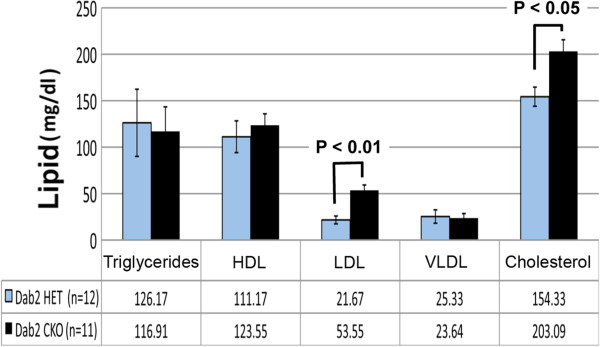
**Increased cholesterol and low density lipoprotein (LDL) levels in Dab2 deficient mice.** Blood was collected for lipid analysis from 6 month old littermate control and Dab2 deficient mice with *dab2* (df/df);Sox2-Cre genotype. The values are reported as the average +/- standard deviation. Student T-test indicates the significance, P < 0.05 for total cholesterol and P < 0.01 for cholesterol from LDL fraction.

In cultured cells, Dab2 co-localizes with the LDL receptor in clathrin-coated pits [36] and modulates LDL uptake [45]. Thus, we inquired about the possible role of Dab2 in LDL uptake in vivo by comparing serum cholesterol levels between littermates of Dab2 sufficient (*dab2* (+/fl);Sox2-Cre) and deficient (*dab2* (df/fl);Sox2-Cre) mice. Serum total cholesterol level was slightly but consistently elevated in Dab2-null over heterozygous mice (Figure 5). A small difference in serum cholesterol was found consistently in 4 independent assays using small (2 to 3 mice for each genotype) groups of mice over a period of several months. In a more deliberate effort to compare serum cholesterol in groups of control and mutant mice shown in Figure 5, we found the small increase to be statistically significant (p < 0.05). Dab2 deficiency influenced mainly the LDL level (p < 0.01), and had little effect on circulating triacylglycerol, HDL, and VLDL levels.

In our breeding scheme of using male *dab2* (+/df);Sox2-Cre and female *dab2* (fl/fl) parents, no *dab2* wildtype littermates were generated to be used as controls. However, the serum lipid profiles of *dab2* (fl/fl) mice were found identical to those of heterozygous and containing Cre transgene (*dab2* (+/df); Sox2-Cre), indicating there was no dosage effect of *dab2* gene nor an impact of Cre expression on cholesterol metabolism.

Thus, the results support the findings from cell culture studies [36,45] that Dab2 is involved in LDL receptor endocytosis and LDL uptake, and suggest that Dab2 has a physiological role in cholesterol metabolism.

### Analysis of dab2-null embryoid bodies and embryos for adaptor proteins

**Figure 6:**
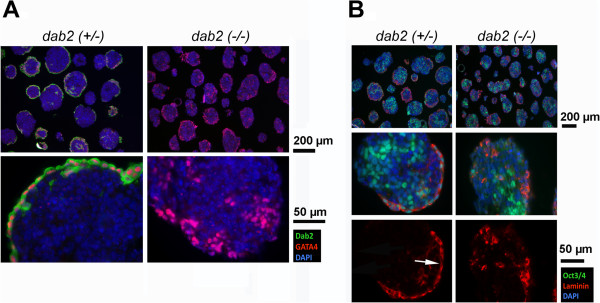
**Endoderm disorganization of Dab2 knockout embryoid bodies.** Clones of ES cells isolated from blastocysts derived from matings between *dab2* (+/df) parents were genotyped by PCR and allowed to form embryoid bodies in suspension cultures for 5 days. Representative examples of heterozygous *dab2* (+/-) and homozygous *dab2* (-/-) embryoid bodies were analyzed by immunofluorescence microscopy. **(A)** Sections were stained for the presence of Dab2 (green), endoderm marker GATA4 (red), and counterstained with DAPI (blue). Merged images at low (top panels) and higher magnification (lower panels) are shown. **(B)** Sections were stained for the pluripotent marker Oct3/4 (green), laminin (red) to indicate primitive endoderm epithelia, and counter-stained with DAPI (blue). Merged images are shown at the top (low magnification) and middle (higher magnification) panels. Laminin staining (red) alone from the corresponding middle panels is presented at the bottom, and the presence of a thin basement membrane underlying the endoderm epithelium in the wildtype embryoid bodies is indicated by an arrow, and no such basement membrane layer was observed in the *dab2* null embryoid bodies.

We generated several lines of both *dab2* heterozygous (+/-) and homozygous (-/-) mutant ES cells from blastocysts for further study. Previously, embryoid bodies derived from ES cells in which Dab2 expression was suppressed by shRNA were defective in the organization of primitive endoderm layer [18]. The ability of *dab2*-null ES cells to form embryoid bodies was tested. In several Dab2 mutant clones analyzed, primitive endoderm differentiation occurred, but the Gata4-positive endoderm cells failed to form an epithelial layer on the surface of the embryoid bodies (Figure 6A). In heterozygous and wildtype controls, a layer of laminin-positive basement membrane was observed (indicated by arrows in Figure 6B), indicating the formation of an epithelium. In Dab2-negative embryoid bodies, the differentiated primitive endoderm cells were positive for laminin; however, neither an endoderm epithelium nor a basement membrane layer was evident (Figure 6B).

**Figure 7:**
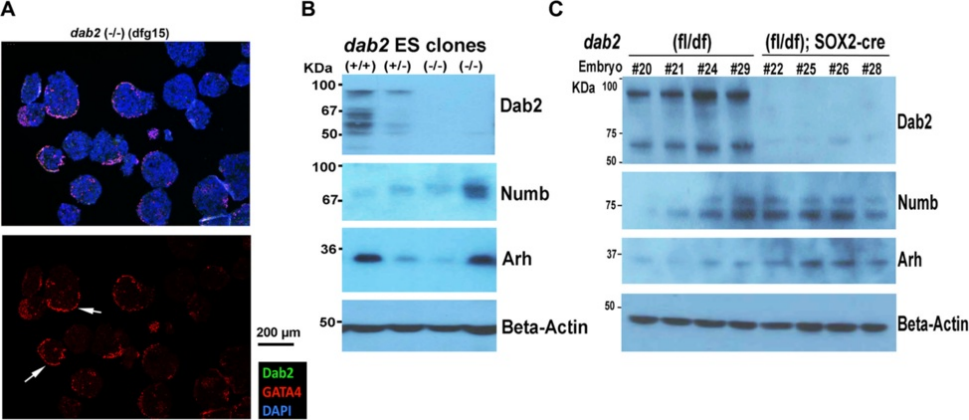
**Mild defective positioning phenotype in embryoid bodies and compensatory expression of Arh and Numb in Dab2 null ES cells and embryos. (A)** In some of the *dab2* (-/-) ES clones (such as clone dfg15), a monolayer epithelial structure is seen at some of the surface of embryoid bodies (indicated by arrows), as shown by an example of a section stained with Dab2 (green) (no signal in this image), GATA4 (red), and DAPI (blue). **(B)** Western blot shows that Arh and Numb were variably expressed in embryoid bodies derived from several ES cell clones of different *dab2* genotypes. **(C)** The expression of Dab2, Arh, and Numb adaptors proteins was analyzed by Western blotting of E9.5 embryos from matings between female *dab2* (fl/fl) and male *dab2* (+/df); Sox2-Cre. The embryos were harvested, dissected free of extraembryonic tissues, lysed and denatured in SDS buffer, and probed for Dab2, Arh, and Numb. The extraembryonic tissues were used for genotyping.

Nevertheless, phenotypic variations were observed among the five *dab2* (-/-) ES clones isolated. A clone of *dab2* (-/-) ES cells (dfg15) was observed to have a mild positioning phenotype, in that some monolayer endoderm epithelial structures were observed in a portion of embryoid bodies derived from this clone (Figure 7A, arrows). We suspect that additional endocytic adaptors with activity similar to Dab2, such as Numb and Arh [31], might be able to substitute for Dab2 function and rescue the endoderm disorganization in a subset of Dab2 mutant embryoid bodies. Indeed, we found that Numb and Arh proteins were expressed in differentiated ES cells in embryoid bodies, and the expression varied among clones (Figure 7B). Compared to other ES clones, the cells of the *dab2* (-/-) clone dfg15 had higher Arh and Numb expression. Additionally, expression of Arh and Numb appeared to change with passage in culture. Thus, the compensatory expression of Numb and/or Arh may rescue primitive endoderm disorganization in embryoid bodies to some degree when *dab2* gene is deleted. Future experiments using combinative deletions or forced overexpression of the adaptors will be needed to test this possibility.

We further determined if compensatory expression of Arh and Numb adaptor proteins occurred in *dab2* knockout embryos. E9.5 embryos from matings between female *dab2* (fl/fl) and male *dab2* (+/df);Sox2-Cre were harvested, and the entire embryo propers dissected free of extraembryonic tissues were analyzed by Western blotting (Figure 7C). Although some variation in levels of Numb and Arh expression were visible in *dab2* heterozygous E9.5 embryos, global expression of Numb and Arh proteins was consistently elevated in the four *dab2*-deleted embryos analyzed (Figure 7C), suggesting there was compensatory expression of Arh and Numb in the absence of Dab2 during embryonic development. Dab1, the other *Drosophila* Disabled ortholog, was undetectable in ES cells or E9.5 embryos, either wildtype or mutant. Involved in neuronal migration and with a brain-restricted expression [4], Dab1 likely has little function or expression overlap with Dab2.

## Discussion

The current *dab2*-conditional deletion mouse model has verified the critical role of Dab2 in the organization of the primitive endoderm [17,18]. However, normal development following its deletion in the embryo proper indicates that the previously suggested roles of Dab2 in the regulation of signaling pathways in unchallenged physiological situations are either very subtle or redundant in vivo.

### Mechanism of epithelial organization and the embryonic lethal phenotype of dab2 knockout mice

The current study confirms the previous observations that Dab2 deficiency leads to disorganization of the extraembryonic endoderm [17,18]. Moreover, we observed primitive endoderm disorganization at E4.5, a stage that immediately follows blastocyst implantation. The extraembryonic endoderm cells are present in Dab2-deficient embryos, but intermingled with the epiblast cells instead of forming a surface epithelial layer. This phenotype of Dab2-deficient embryos prompts us to describe *dab2* as an “epithelial surface positioning gene” [18], in parallel to the manner in which *dab1* is referred to as a “neuronal positioning gene” [4]. The proposed mechanism is that Dab2 mediates directional endocytic transport in the endoderm epithelial cells, leading to the establishment of an apical polarity [18,46]. Previously, it was considered that the highly adhesive embryonic cells sort to inside and less adhesive cells sort to the periphery [47], according to the Differential Adhesion Hypothesis [48,49]. However, in the case of the mouse primitive endoderm, the ability to establish apical polarity rather than differential adhesive affinity determines the anchoring of the primitive endoderm layer at the surface [46]. Thus, deletion of *dab2* leads to the loss or reduced trafficking of cell surface adhesion molecules, consequently a reduced ability to establish a polarity, and finally the failure of primitive endoderm cells to position at the surface [18,46].

Additionally, deletion of *c-fos* can partially rescue endoderm disorganization in the Dab2-deficient embryos [22]. Dab2 can negatively modulate c-Fos expression [19], and c-Fos can regulate epithelial polarity [50]. Thus, Dab2 may also impact endoderm cell organization through its influence on the MAPK/c-Fos signaling pathway. Although, it was reasoned previously that additional activities of Dab2 other than suppression c-For might impact organogenesis [22], the current result indicates Dab2 developmental requirement is restricted to extraembrynic endoderm. However, it is still possible that Dab2 expression within extraembryonic endoderm may influence embryonic development by a non-cell autonomous mechanism.

### Lack of developmental phenotypes in dab2 mosaic mutant mice

Considering that the loss of Dab2 in primitive endoderm leads to the disruption of epithelial organization and a severe phenotype in early embryos, we were perplexed that *dab2* conditional knockouts generated using either Meox2-Cre or Sox2-Cre showed no developmental defects and had relatively mild adult phenotypes. One possibility is that remnant Dab2-positive cells in the mosaic *dab2* mutant mice are sufficient for essential developmental functions. Alternatively, expression of endocytic adaptor proteins such as Numb and Arh, which have similar structures and functions as Dab2 [31], may compensate when *dab2* is deleted. Based on PCR genotyping of tail tissues and immunostaining of kidney, we estimated that around 5-10% of the cells still contain undeleted *dab2* gene in Meox2-Cre conditional knockout. Conversely, the “fl” allele was undetectable in Sox2-Cre knockout lines, yet the percentage of Dab2-positive cells could be higher in the earlier stages of embryonic development. Sox2 is expressed in and essential for the trophoblast extraembryonic lineage [51,52]. However, Sox2-Cre-mediated deletion of *dab2* gene did not affect trophectoderm development, thus Dab2 is also not essential in at least a subset of the trophoblast lineage. Although the *dab2* gene can be deleted in the majority of somatic cells without affecting embryonic development, we considered the possibility that a miniscule fraction of Dab2-positive cells may be sufficient to fulfill its potentially critical roles. Nevertheless, we did not detect any remaining *dab2* allele in E9.5 Sox2-Cre conditional knockout embryos. Most likely, compensatory expression of Dab2-like proteins such as Arh and Numb accounted for a mild phenotype and the actual formation of primitive endoderm structures observed in some of the Dab2-deficient embryoid bodies.

### Dab2 mediates endocytosis and contributes to lipoprotein metabolism in vivo

Although Dab2-deficient mice were overtly normal, mutant mice did have several physiological differences, such as excessive excretion of plasma proteins in urine and increased serum cholesterol and LDL. These physiological phenotypes might be attributable to the adaptor activity of Dab2 for megalin and LDL receptor, respectively. Another well-documented Dab2 binding ligand is integrin [34,35]. Though an impact of Dab2 on cell migration was observed in cultured cells, we found no related physiological phenotype in the mutant mice. Dab2 also has been reported to have many other activities, but these phenotypes were also not readily observable in the mutant mice.

In the case of LDL receptor endocytosis, the increased cholesterol and low density lipoprotein levels in Dab2 deficient mice was small but readily detectable. The increased serum cholesterol and LDL levels was likely due to the absence of Dab2 protein in mediating uptake of LDL particles from circulation by Dab2-mediated endocytosis. The results support the findings in cell culture studies [36,45] that Dab2 is involved in LDL receptor endocytosis and LDL uptake, and suggest that Dab2 has a physiological role in cholesterol metabolism. Normally, mice have a relatively low level of serum LDL. Possibly, the difference in serum cholesterol and LDL levels would be more significant in Dab2-deficient mice when the animals are placed on a high fat diet. The impact of Dab2 on cholesterol metabolism, the cell types affected, and comparison with LDL receptor and Arh mutant mice, will be interesting topics of future investigations.

Likely, as an endocytic adaptor for several cell surface receptors, Dab2 may influence the endocytosis and secretion of multiple ligands, and may regulate multiple signaling pathways. The function of Dab2 is pleiotropic, although it may serve as a fine tune regulator of signaling and an endocytic adaptor with overlapping specificity with other adaptors, but the impact of its deletion is mild when observed in normal physiological setting. However, additional phenotypes and Dab2 functions may be observed in deeper analysis of the Dab2 deficient mice, and also when placing the mutant mice in a challenged situation.

### Cellular actions of Dab2 in signaling and cell adhesion

Dab2 has been implicated in regulating signaling pathways, including the Ras/MAPK/c-Fos [19-22], the TGF-beta [15,23,24], and the Wnt pathways [25-28]. Dab2 also participates in the endocytic recycling of E-cadherin [18] and integrins [35] and thereby influences cell adhesion and mobility. It is plausible that Dab2 affects these diverse cellular signaling pathways due to its role in endocytosis and trafficking; however, whether these activities are physiologically significant has not been resolved previously. The Ras/MAPK pathway [53-59], the TGF-beta pathway [60], the Wnt pathway [61,62], E-cadherin [63,64], and integrins [65] are all critical in embryonic development and their perturbations impair the process. Since Dab2 deletion in the embryo proper minimally impacts embryonic development, we would argue that the majority of the proposed Dab2 functions generated from these in vitro studies have little relevance in vivo. Dab2 may fine-tune the activities of these signaling pathways, but the effects are subtle and not readily observable in the normal physiology of whole animals.

### Loss of Dab2 in epithelial disorganization and cancer

One common observation in both early embryonic development and carcinogenesis is that loss of Dab2 leads to epithelial morphological transformation. The loss of Dab2 correlates well with morphological changes, specifically the transition from a simple epithelial monolayer to a multiple layered neoplasm in ovarian cancer [66]. This is remarkably reminiscent of the disorganization and loss of surface position of primitive endoderm in *dab2*-deficient embryos and embryoid bodies. Dab2-mediated directional endocytic trafficking leads to the generation of an apical-basal epithelial polarity, and polarity plays critical role in surface positioning [18]. Thus, loss of Dab2 results in epithelial depolarization and subsequent disorganization, as found in ovarian carcinomas and early embryos [17,18,39,66]. However, ovarian surface epithelia are monolayered and intact in *dab2*-deficient mice (not shown), indicating the loss of Dab2 alone is insufficient to induce epithelial disorganization. Unlike the primitive endoderm epithelium in rapidly growing embryos, mature epithelia in adults most likely possess additional mechanisms to ensure stable architecture.

## Conclusion

The current results in the study of a new *dab2* conditional mutant allele are consistent with the prior report that *dab2* deletion leads to an early embryonic lethality at E5.5 [17,18]. This lethality occurs much earlier than the other report of mutants derived from a previously reported conditional allele [38]. Consistent with our prior conclusion [18,19], the current analysis of both embryos and embryoid bodies indicates that loss of Dab2 leads to disorganization of primitive endoderm and subsequent extraembryonic endoderm, although the differentiation to endoderm lineage is not impaired. Thus, the current study clarifies the discrepancy in embryonic phenotypes described for the two previous knockouts [17,38].

Conditional deletion indicated that Dab2 is dispensable for organ development, when the vast majority of the embryonic cells are *dab2* null. A possibility is that additional endocytic adaptors such as Arh and Numb partially compensate for Dab2 loss. However, Dab2 has a physiological role in the endocytosis of lipoproteins and cholesterol metabolism.

## Methods

### Construction of the dab2 homologous recombination targeting vector and conditional mutant mice

Using the mouse Dab2 cDNA as a probe, three clones (λ20, λ24, and λ28) of mouse genomic DNA containing the *dab2* gene were isolated from a 129/Sv genome library in λZap (Stratagene) [67]. The λ24 fragment, spanning exons 2 to 11 of the *dab2*, was cloned at the *Eco* RI site of pBSKS, and was used as a template for PCR amplifications. A 5.8 kb genomic fragment covering exons 5 to 9 was PCR-amplified with a Not I-anchored sense primer (5′-ATG GAT GCG GCC GCT CCC GGA AAT GGT TAC-3′) and antisense primer (5′-ATG ATG GAT CTT TGG TTG TTG T-3′). The resulting PCR fragment was recloned into Apa I/Not I restricted pK11/pM30 Frt-PGKNeo-Frt-LoxP-pBSSK plasmid (from Dr. David W. Martin, Emory University) which was first modified by cloning a polylinker (*Apa* I-*Sac* I-*Not* I-*Stu* I-*Kpn* I) at *Apa* I/*Kpn* I sites with the sequence of 5′-CAT GAG CTC AGG CGG CCG CAT AGG CCT AAG GTA C-3′. A 1.7 kb genomic fragment containing exon 2 was PCR-amplified with *Cla* I-anchored sense primer (5′-ATC GAT CTG CAG TGA GGA TCC TGA ATA CTA TCT CTC GGT ACT-3′) and *Sal* I-*Sac* I-anchored antisense primer (5′-ATC GAT GTC GAC TGA GAG CTC CAC ATT CTG CTA ATA TGT CAT C-3′) and inserted into *Cla* I/*Sal* I restricted BstLox2 vector (also provided by Dr. D.W. Martin) upstream of the LoxP site near the T7 RNA promoter. A 2.8 kb fragment containing exons 3 and 4 was PCR-amplified with Sac II-anchored sense primer (5′-ATC GAT CCG CGG GAA TGA ATC CTA CCA TGG-3′) and *Eco* RV-anchored antisense primer (5′-ATC GTA GAT ATC AGC CTG CCA GAG CTG GAG-3′) and recloned into *Eco* RV/*Sac* II restricted BstLox2 vector downstream of the LoxP site. The subcloned fragment of 4.4 kb containing exon 2-Loxp-exon 3 and 4 of the *dab2* gene was then digested with Sac I and Sac II and recloned into pK11/pM30 Frt-PGKNeo-Frt-LoxP-exons 5 to 9 to generate the final targeting construct. Each PCR fragment generated was amplified using cloned-Pfu DNA polymerase (Stratagene), which has a proofreading activity that results in high-fidelity DNA replication. At each cloning step, several clones were verified by restriction analysis and sequencing. The targeting vector was linearized with *Sac* I and *Avr* II to obtain an 11.7 kb fragment that was electroporated into RW4 ES cells.

The selection, screening, and blastocyst injection to produce chimeric mice were performed with assistance from the institutional transgenic mouse facility according to standard procedures. Neomycin resistant ES cell clones were screened for homologous recombination by PCR using discriminating primers spanning sequences both inside and outside the targeting construct. The sequences of the primers are listed: P5′: 5′-CCA GTA CAC CAC GTA AGA AAG-3′; P3′ for WT: 5′-ACA GTC ACT GAT ACC AGC CAA-3′; P3′ for KO: 5-AGT TAT TAG GTC CCT CGA CCT-3′. The fragments from PCR amplification were predicted to be 886 bp for wildtype (WT) and 1714 bp for the recombinant allele. The positive clones were further characterized by PCR to ensure the allele contained all the component of the targeting construct: LoxP 1, 1714 bp (P5′: 5′-CCA GTA CAC CAC GTA AGA AAG-3′; P3′: 5-AGT TAT TAG GTC CCT CGA CCT-3′); LoxP 2, 795 bp (P5′: 5′-ACA TTA TAC GAA GTT ATT CGA GG-3′; P3′: 5′-ATC ACA GTT GGC GTC ATA ACA A-3′); Frt1, 635 bp (P5′: 5′-CAC CTG ATC TGA CTG TGG TT-3′; P3′: 5′-AGA GAA TAG GAA CTT CGG CC-3′); Frt2, 837 bp (P5′: 5′-ACT ATA GGA ACT TCG TCG ACC-3′; P3′: 5′-ATC ACA GTT GGC GTC ATA ACA A-3′); Neo, 617 bp (P5′: 5′-GGC ATT CTC GCA CGC TTC AA-3′; P3′: 5′-CTT GAG CCT GGC GAA CAG TT-3′).

Two selected recombinant ES clones, #28 and #121, were used for blastocyst injection to establish mutant lines. The neo locus flanked by Frt sites was then excised by crossing with FLPeR mice [40,41]. Two lines of *dab2* flox conditional knockout mice (lines 239 and 270) were established from the two independent ES clones and the mouse colonies were maintained in the C57BL/6 J background. The two lines gave identical phenotypes and were not distinguished here unless specifically mentioned.

### Mouse strains and husbandry

Flp expression mice (129S4/SvJaeSor-Gt(ROSA)26Sortm1(FLP1)Dym/J) [40,41], Meox2-Cre mice (B6.129S4-Meox2tm1(cre)Sor/J) [44], and Sox2-Cre mice (Tg(Sox2-cre)#Amc/J) [42,68] were purchased from Jackson Labs.

Control and mutant mice were kept in the institutional mouse facility as inbred colonies by pairing parents of selected genotypes. Upon weaning, the mice were genotyped and separated into experimental and control groups accordingly. The mice were caged in barricaded viral pathogen-free rooms under a 12-hour light–dark cycle, with free access to food and autoclaved water. For dietary studies, the mice were kept on a 5 K20 chow (LabDiet: 17% protein, 10% fat, 2.5% fiber, 8% ash, and 3.5% added minerals). All procedures for experiments using animals were reviewed and approved by the University of Miami Miller School of Medicine Institutional Animal Care and Use Committee and followed National Institutes of Health guidelines.

### Measurement of serum lipids

Mice were fasted for 6 hours and then anesthetized before blood was collected by cardiac puncture. Blood samples were allowed to clot for 15 min and centrifuged for 10 min in a bench top centrifuge. The serum supernatants were collected and stored at -80°C until use. Lipid analysis was performed by the University of Miami Comparative Pathology Laboratory using a Vitros 250 chemistry analyzer (manufacturer: Johnson & Johnson). Cholesterol, triglycerides, and high-density lipoprotein (HDL) levels were measured colorimetrically. Low-density lipoproteins (LDL) and very low-density lipoprotein (VLDL) levels were derived from the following formula: VLDL = 1/5 of triglycerides; LDL = total cholesterol minus HDL and VLDL).

### Derivation of dab2 null ES cells and the formation of embryoid bodies

Blastocysts were harvested at E3.5 after timed-matings between *dab2* (+/-) parents. The blastocysts were individually cultured in single wells of 24-well tissue culture plates previously coated with irradiated murine embryonic fibroblasts (MEF). The blastocysts were allowed to hatch and attach to the fibroblast feeder layer. Cells from the outgrowth of blastocysts were expanded and these clones were established and characterized to confirm as ES cells, as described previously [69]. Established ES cells were maintained in a pluripotent state by culturing on an irradiated MEF feeder layer in media supplemented with 1000 U/ml of recombinant LIF (ESGRO, Chemicon International).

To produce embryoid bodies, approximately 6 × 10^6^ ES cells were suspended in 10 ml of medium lacking LIF on non-adhesive Petri dishes and allowed to aggregate for 5 to 7 days. After the first two days, 5 ml of fresh medium was added to the plates. Afterward, the medium was then changed every other day by collecting the cell aggregates with brief centrifugation (2,000 *g* for 1 min) and re-suspending the aggregates in fresh medium by gently pipetting the aggregates. In some experiments, retinoic acid (1 μM) was added in the medium to enhance endoderm differentiation. At the end of each experiment, the cells aggregates were collected and processed for Western blot or histology analysis. The starting concentrations of ES cells were adjusted to ensure the resulting embryoid bodies are in a size range of 20–100 μm, which is comparable to the size of embryo cross sections at E5.5 stage.

### Genotyping

At earlier stages (E5.5 and E4.5), *dab2* genotyping was performed by examining the morphology and/or Dab2 immunostaining of the embryos to distinguish *dab2* (+/+) or (+/-) from (-/-) genotypes.

For embryos at later stages (E9.5) and adult mice, DNA was extracted from entire embryo proper, yolk sacs, or tail biopsies. PCR amplification with primers 5′-TAC AGG CAT CCC CAT TTT TG -3′, 5′-TGC CAC CTA CAA GGA AGG AC-3′, and 5′-ACA GGC TGT GCA GTC TCG TA-3′ generated amplicons of 180 bp (*dab2* wildtype, or wt), 284 bp (flox, or fl), and 529 bp (delta flox, or df). Cre alleles were amplified using the generic Cre primers: 5′-CCT GGA AAA TGC TTC TGT CCG -3′ and 5′-CAG GGT GTT ATA AGC AAT CCC-3′ to generate a 400 bp fragment.

### Histology, histochemistry, and immunofluorescence microscopy

Embryos from timed matings were harvested either while in utero (up to E7.5) or as individually dissected embryos (E9.5). Part of the later stage embryos was used for genotyping. The collected specimens were processed by formalin fixation and paraffin embedding. The archived tissues were then sectioned and processed for histology and immunofluorescence microscopy.

Primary antibodies used in this study were: mouse monoclonal anti-Dab2 (BD Transduction Laboratories); rabbit polyclonal anti-Dab2 [18]; rabbit polyclonal anti-Gata4 antibodies (Santa Cruz Biotechnology, Inc.); rabbit pan anti-laminin (Abcam); rabbit polyclonal anti-GATA6 [70]; mouse monoclonal anti-Oct3/4 (Santa Cruz Biotechnology); mouse monoclonal anti-Arh (Santa Cruz Biotechnology, Inc.); goat polyclonal anti-Numb (Abcam), and mouse monoclonal anti-beta-actin (BD Transduction Laboratories). DAPI (4′-6-diamidino-2-phenylindole) was used as a generic nuclear counterstain and applied at the terminal stages of the procedure.

Conventional wide-field microscopy was performed on an inverted Zeiss AxioObserver Z1 operated by Axio Vision 4.8 software. Images were acquired digitally with a monochrome Zeiss AxioCam MRm CCD camera.

## Competing interests

The authors declare that they have no competing interests.

## Authors’ contributions

All authors participated in the initial planning and development of the experimental rationale. RM designed most of the experiments, maintained mouse colonies, made ES cells, and performed embryo dissections and embryoid body analysis. KQC performed immunohistochemistry on embryos. WT contributed to serum cholesterol analysis and Western blot. ERS also contributed to Western blot and other analysis, and especially contributed to editing and writing of the manuscript. XXX coordinated and supervised the project and wrote the first draft of the manuscript. All authors were involved in discussion, several revisions, and editing of the manuscript, and all read and approved the final version.

## Supplementary Material

Additional file 1: Table S1. Partial breeding record for *dab2* (fl/+) mice from line 239. **Table S2.** Partial breeding record for *dab2* (fl/+) line 270. Click here for file
