# Supplementary material for: Differential requirement for Dab2 in the development of embryonic and extra-embryonic tissues
Source: BMC Dev Biol. 2013 Oct 29;13:39. doi: 10.1186/1471-213X-13-39 (PMC3924344; doi:10.1186/1471-213X-13-39)
Supplement: Additional file 1: Table S1. — Partial breeding record for dab2 (fl/+) mice from line 239. Table S2. Partial breeding record for dab2 (fl/+) line 270. [file 1471-213X-13-39-S1.doc]

**Supplementary Table 1. Partial breeding record for *dab2* (fl/+) mice from line 239.**

| ***dab2* (fl/fl)** | | ***dab2* (fl/+)** | | ***dab2* (+/+)** | |
| --- | --- | --- | --- | --- | --- |
| **male** | **female** | **male** | **female** | **male** | **female** |
| 96, 117, 123, 145, 148, 152, 160 | 98, 100, 104, 106, 107, 120, 121, 126, 128, 134, 163, 170 | 82, 84, 85, 90, 91, 101, 113, 115, 116, 124, 132, 146, 147, 157, 158, 159, 165, 173 | 81, 94, 99, 103, 105, 118, 119, 122, 127, 133, 135, 136, 151, 154, 155, 161, 162, 164, 167, 171, 172 | 78, 79, 83, 86, 92, 97,102, 114, 125, 129, 130, 131, 143, 144, 149, 150, 153, 156, 166 | 80, 87, 88, 89, 93, 95, 168, 169 |
| n = 7 | n = 12 | n = 18 | n = 21 | n = 19 | n = 8 |
| Σ = 19 (22.4%) | | Σ =39 (45.8%) | | Σ = 27 (31.8%) | |
| Σ = 85 (100%) | | | | | |

**Notes:** The mice produced from the matings between heterozygous parents of line 239 were traced by the number indicated and were categorized according to genotypes and sex. The ratio of *dab2* (fl/fl), (+/fl), and (+/+) is 1:2:1.4.

**Supplementary Table 2. Partial breeding record for *dab2* (fl/+) line 270.**

| ***dab2* (fl/fl)** | | ***dab2* (fl/+)** | | ***dab2* (+/+)** | |
| --- | --- | --- | --- | --- | --- |
| **male** | **female** | **male** | **female** | **male** | **female** |
| 45, 46, 83, 86, 89, 94, 96, 105, 111 | 70, 72, 73, 85, 91, 98, 106, 112, 113, 114 | 47, 54, 55, 58, 64, 65, 74, 75, 76, 82, 84, 88, 93, 103, 104 | 38, 39, 41, 48, 49, 52, 56, 57, 59, 60, 61, 62, 63, 67, 69, 77, 78, 79, 81, 92, 97, 99, 107, 109, 110 | 44, 87, 95, 102 | 40, 42, 53, 66, 68, 71, 80, 90, 108 |
| n = 9 | n = 10 | n = 15 | n = 25 | n = 4 | n = 9 |
| Σ = 19 (26.4%) | | Σ = 40 (55.6%) | | Σ = 13 (18.0%) | |
| Σ = 72 | | | | | |

**Notes:** The mice produced from the matings between heterozygous parents of line 270 were traced by the number indicated and were categorized according to genotypes and sex. The ratio of *dab2* (fl/fl), (+/fl), and (+/+) is 1:2.1:0.68.
